# Photo-Switchable Sulfonylureas Binding to ATP-Sensitive Potassium Channel Reveal the Mechanism of Light-Controlled Insulin Release

**DOI:** 10.1021/acs.jpcb.1c07292

**Published:** 2021-11-26

**Authors:** Katarzyna Walczewska-Szewc, Wieslaw Nowak

**Affiliations:** Faculty of Physics, Astronomy and Informatics, Nicolaus Copernicus University in Torun, ul. Grudziadzka 5, 87-100 Torun, Poland

## Abstract

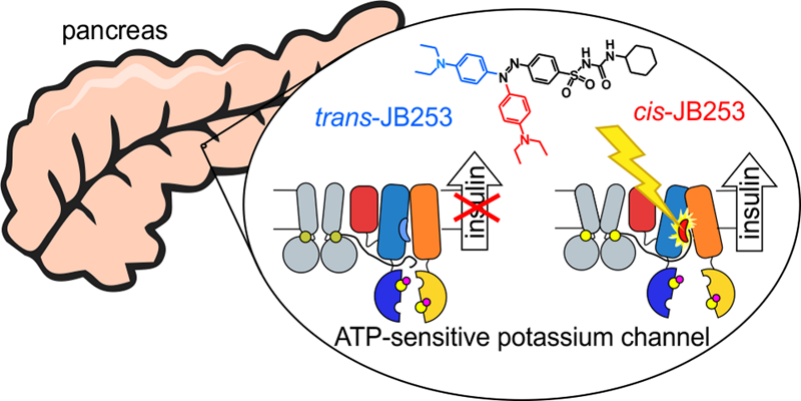

ATP-sensitive potassium (KATP) channels are present in numerous organs, including the heart, brain, and pancreas. Physiological opening and closing of KATPs present in pancreatic β-cells, in response to changes in the ATP/ADP concentration ratio, are correlated with insulin release into the bloodstream. Sulfonylurea drugs, commonly used in type 2 diabetes mellitus treatment, bind to the octamer KATP channels composed of four pore-forming Kir6.2 and four SUR1 subunits and increase the probability of insulin release. Azobenzene-based derivatives of sulfonylureas, such as JB253 inspired by well-established antidiabetic drug glimepiride, allow for control of this process by light. The mechanism of that phenomenon was not known until now. In this paper, we use molecular docking, molecular dynamics, and metadynamics to reveal structural determinants explaining light-controlled insulin release. We show that both *trans-* and *cis-*JB253 bind to the same SUR1 cavity as antidiabetic sulfonylurea glibenclamide (GBM). Simulations indicate that, in contrast to *trans-*JB253, the *cis-*JB253 structure generated by blue light absorption promotes open structures of SUR1, in close similarity to the GBM effect. We postulate that in the open SUR1 structures, the N-terminal tail from Kir6.2 protruding into the SUR1 pocket is stabilized by flexible enough sulfonylureas. Therefore, the adjacent Kir6.2 pore is more often closed, which in turn facilitates insulin release. Thus, KATP conductance is regulated by peptide linkers between its Kir6.2 and SUR1 subunits, a phenomenon present in other biological signaling pathways. Our data explain the observed light-modulated activity of photoactive sulfonylureas and widen a way to develop new antidiabetic drugs having reduced adverse effects.

## 1 Introduction

The absorption of light by chemical compounds may lead to substantial conformational changes, which could be further exploited to steer biological processes. The typical example is isomerization of 11-*cis*-retinal in rhodopsin, enabling vision in animals and humans^1^ and being exploited in optogenetics.^2^ The usage of light to control ion channels offers excellent spatial and temporal resolution in investigations of neural systems.^3^ That concept is also a basis of photo-pharmacology, which brings new possibilities in drug design.^4,5^ Incorporating photosensitive components into a drug molecule allows for precise control of drug–target interactions in an irradiation-dependent manner. Should the drug activity be limited to a specific area and a time span, unwanted adverse or side effects might be reduced.^6^

The concept of using light to control the action of drugs has been explored intensively over the past.^4,5,7−11^ Light-sensitive molecules were considered as promising tools in the treatment of cancer (photo-activated chemotherapy),^4^ bacterial infections (photoactive antibiotics),^7^ Alzheimer’s disease,^8^ or degenerative retinal diseases.^9^ Computational methods were successfully used in studies of such systems.^12,13^

**Figure 1:**
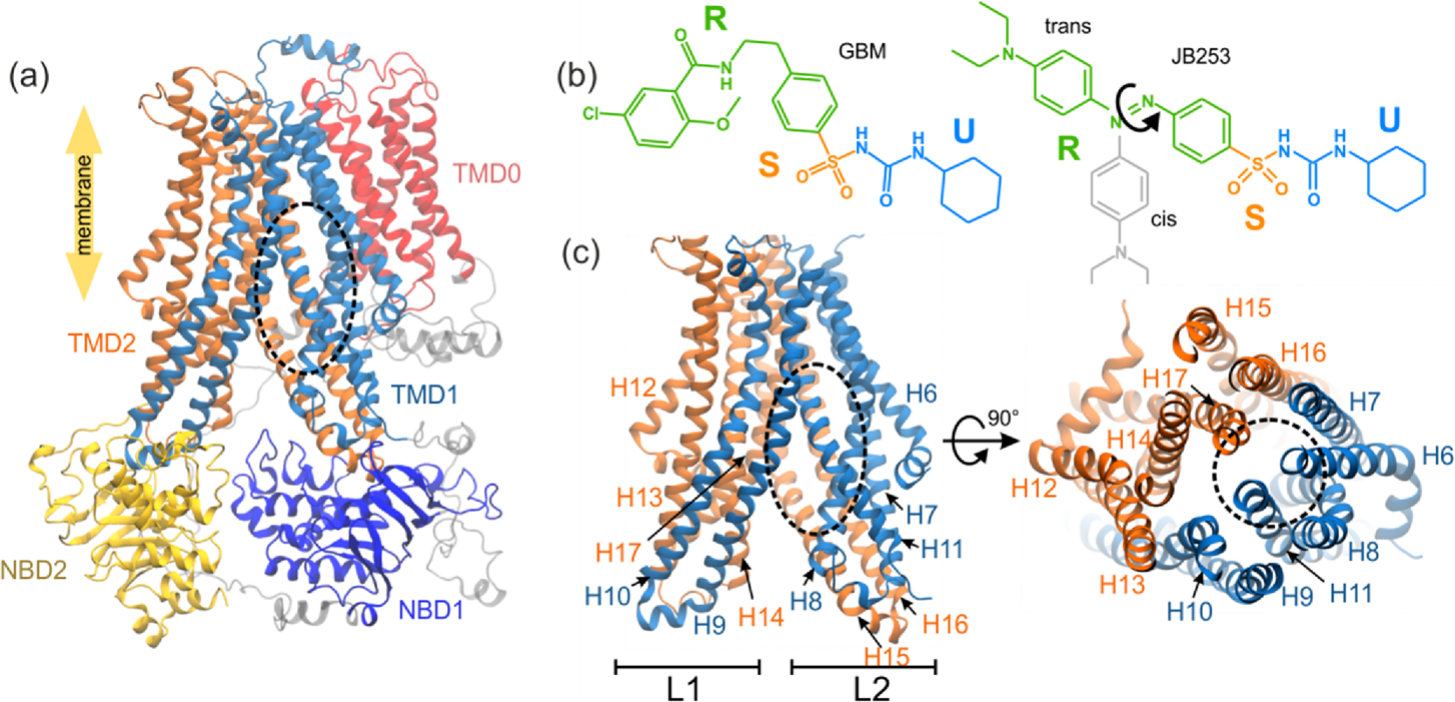
Model of SUR1 used in simulations (the binding site of glibenclamide (GBM) is shown as a black dashed oval, parts that are unresolved in the original PDB structure are shown in gray, and NBD1 and NBD2 denote the nucleotide binding domains, whereas TMD0, TMD1, and TMD2 stand for the SUR transmembrane domains) (a); structures of the ligands used in this study: GBM and JB253 in cis and trans conformations (b) (blue, orange, and green colors represent the U, S, and R functional parts of ligands, respectively); the transmembrane part of the SUR1 ABC domain in side and top views (c).

In this work, we focus on the possible usage of photoactive sulfonylurea drugs to treat type II diabetes (T2DM). Experiments performed by Broichhagen et al.^10,14^ showed that photo-switchable sulfonylurea derivatives (see Figure 1b) could effectively control insulin release from isolated murine islets. The molecular basis of such promising results is not known. This work provides data explaining the initial stage of light-induced insulin release from pancreatic β-cells treated by specific photoactive sulfonylureas.

Sulfonylurea drugs (SUs) have been used to control the blood glucose level in T2DM patients since 1956.^15^ Currently, we know that SUs, e.g., glibenclamide (GBM, also called glyburide), glipizide, gliclazide, glimepiride, and other secretagogues such as glinides, inhibit the action of ATP-sensitive potassium (KATP) channels present in the pancreas and other human tissues. KATPs control many physiological processes, such as hormone secretion and vasodilatation. KATP channels, which are of interest here, are hetero-octameric structures composed of four pore-forming Kir6.2 subunits and four regulatory sulfonylurea receptor subunits (SUR1) (see Figure S1 for details). By binding to SUR1 (Figure 1a), sulfonylureas stop or reduce K^+^ ions efflux from the β-cell. It leads to cell membrane depolarization and the opening of voltage-gated calcium channels.

The influx of Ca^2+^ ions drives a downstream cascade that ultimately leads to insulin secretion from β-cells.^16^ Numerous mutations in either the Kir6.2 or SUR1 subunits are linked to neonatal diabetes or congenital hyperinsulinism.^17,18^ Although SUs, known for the past 50 years, are still used in 25% of cases of T2DM treatment, they have many drawbacks.^19^ First of all, since their oral administration regime is based on estimations and not on the actual level of the blood glucose measurements, SUs may provoke episodes of hyperinsulinemia. Moreover, KATP channels are present in various tissues, including the skeletal muscle,^20^ visceral and vascular smooth muscle,^21^ heart, and brain.^22^ Inhibition of the off-target channels can lead to serious side effects, such as elevated cardiovascular disease risk,^23^ and can induce 1–4 kg weight gain. The exquisite spatiotemporal control conferred by newly developed photoactive compounds could potentially open up new ways for the T2DM treatment.

Broichhagen et al. described a photo-switchable SU, JB253, which reversibly and repeatedly blocks the KATP channel following exposure to violet-blue light.^10^ The photo-responsiveness of their “fourth-generation sulfonylurea” was obtained by introducing the azobenzene moiety into a classical glimepiride drug. Photo-isomerization of the ground state *trans*-azobenzene moiety to the cis form leads to the KATP channel closure. Thermal relaxation restores the trans form of JB253 and is correlated with the re-occurrence of the open form of the KATP channel. In that way, light-controlled insulin release in mice β-cells has been demonstrated.^10^ However, a detailed molecular mechanism of JB253 action remains unknown, and this problem is addressed here.

In this work, we use molecular docking, unbiased and enhanced sampling molecular dynamics (MD) simulations to investigate binding modes of JB253 and a generic SU drug GBM to SUR1. Starting from cryo-EM structures of human^24^ and hamster^25^ KATP and using all-atom MD simulations, we scrutinize global and local structural changes in SUR1 upon GBM and JB253 binding. For JB253, we focus on the possible effects of adopting trans or cis forms on the SUR1 protein structure. We use metadynamics^26^ to calculate the free energy profiles along selected distance coordinates to estimate the probability of inward open (later “open”) and outward open (later “close”) forms of SUR1. These forms are strictly correlated with closing and opening of the KATP channel, respectively.^24,25,27−29^ Our in silico findings demonstrate that both *trans*-JB253 and *cis*-JB253 bind to the same region of SUR1 as the GBM drug does. We show that the light-triggered conformational change of JB253 enhances the probability of SUR1 opening, resulting in an increased probability of KATP closing and insulin release. In short, our modeling shows that the photo-isomerization makes JB253 act as a standard SU drug and confirms the utility of the opto-pharmacological approach to the T2DM problem.

## 2 Methods

### 2.1 Structure Preparation and Ligand Docking

We have chosen to use the structure of human SUR1 (PDB ID: 6C3P) as our initial model. The conformation of the protein was changed from the outward open (here: close) to the inward open (here: open) using the targeted MD method.^30^ Such a method is commonly used to enforce a change in the conformational states of proteins.^31,32^ Details are described in ref (33). The resulting structure overlaps the target, which was hamster open SUR1 (PDB ID: 6BAA). Since there is a large sequence similarity between human and hamster SUR1 (95%, for comparison see SI, Figure S10), using such an open structure as a template for human protein is justified and should not generate serious bias.

The ligands were docked into the open SUR1 structure using Glide 2018.4.^34^ First, GBM and both JB253 isomers were prepared with LigPrep using the OPLS3e force field. The receptor grid for docking was centered in the GBM binding site from PDB ID: 6BAA and was able to accommodate ligands with lengths up to 20 Å. All ligands were docked with standard precision, using flexible ligand sampling (see the SI for further details). We chose the lowest energy pose of each ligand as the starting point for the simulation. Based on energy criteria for *cis*-JB253, a chiral enantiomer P was selected for further modeling.

Using the CHARMM36 force field,^35^ we imported those models into GROMACS 2019.^36^ Each system was embedded into a POPC lipid bilayer and solvated in a 110 × 110 × 170 Å box of TIP3P water, resulting in a molecular system of about 235,000 interacting particles. The net charge of the system was neutralized by the addition of K^+^ and Cl^–^ ions (233 and 238 ions, respectively). The topology parameters and charges of GBM and both forms of JB253 have been obtained from Dr. L. Peplowski (personal communication, details of parametrization, and parameters validation in the SI). The lipid force field was taken from Konrad et al.^63^ The particle-mesh Ewald method with a short-range cutoff of 1.2 nm was applied to treat electrostatics. The force-based cutoff of 1.2 nm was applied to the non-bonded van der Waals interactions. The force field used for docking (OPLS3e) is different than that for MD simulations (CHARMM36) but this does not affect MD results much since we observed that on MD trajectories, ligand positions are maintained in the pocket.

### 2.2 Molecular Dynamics Simulations

#### 2.2.1 Unbiased MD

We use the GROMACS 2019 package for all classical MD simulations. First, all systems were minimized by the steepest descent energy minimization algorithm (1000 steps). Then, we created five replicas of each system. The equilibration procedure was the same for each system: the protein, ligand, and lipid heads were first held fixed while water and lipid tails were allowed to equilibrate for 2 ns. Then, three subsequent MD simulations (2 ns each) were run in the NVT ensemble at 310 K, applying position restraints of 1000, 500, and 100 kJ/mol/nm^2^, respectively, to the protein alpha carbons and the ligand. The fourth step of position restrained simulation (10 ns, 10 kJ/mol/nm^2^ harmonic restrains held on protein Cα and ligand) was carried in the NPT ensemble with a constant pressure of 1 atm maintained using a Berendsen barostat.^37^ Finally, we run an unrestrained production MD of 225 ns in the NVT ensemble, maintaining a constant temperature of 310 K using a v-rescale thermostat.^38^ A 2 fs time step was used, and all bonds to hydrogen atoms were fixed (LINCS).^39^ Once complete, the first 25 ns of each simulation was excluded from further analysis to account for the equilibration of the drug in the SUR1 cavity. The level of equilibration was monitored using RMSD.

#### 2.2.2 Metadynamics

Well-tempered metadynamics^40^ (WTM) was used to enhance the conformational change of opening and closing the NBD domains in SUR1. In this work, we run WTM using the open-source, community-developed PLUMED library,^41^ version 2.4,^42^ patched to GROMACS 2018.4. To investigate the free energy landscape of changing SUR1 from open to close conformation, we chose a collective variable, representing a distance between centers of mass of NBD1 (residues 677 to 739 and 770 to 912) and NBD2 (residues 1337 to 1561). Additional Gaussian-shaped potential with a height of 1.0 and width of 0.5 kcal/mol was deposited every 100 time steps, equivalent to 0.2 ps. A bias factor of 20 was used for well-tempered dynamics. The results represent 170 ns of simulation for each drug. Boundaries were imposed for minimal (25 Å) and maximal (60 Å) distances using flat, bottomed harmonic potentials (WALLS keyword in the PLUMED package). Available cryo-EM structures indicate that such distances are 28.89 and 40.1 Å in the closed and open form of SUR1, respectively. Convergence of the free energy profiles in each system was reached approximately after ∼80 ns. After these time points, the Gaussian height of the bias added to the system during the metadynamics simulations gradually reached zero. The entire distance range was sampled multiple times for each simulation.

### 2.3 Data Post-processing and Visualization

A set of Python scripts for alignment of trajectories, RMSD and RMSF calculations, and a detailed analysis of systems geometry, were written using, among others, NumPy,^43^ SciPy,^44^ and MDAnalysis^45^ modules.

TMD1 and TMD2 transmembrane domains in all trajectories were aligned to the initial frame of the APO simulation.

**Figure 2:**
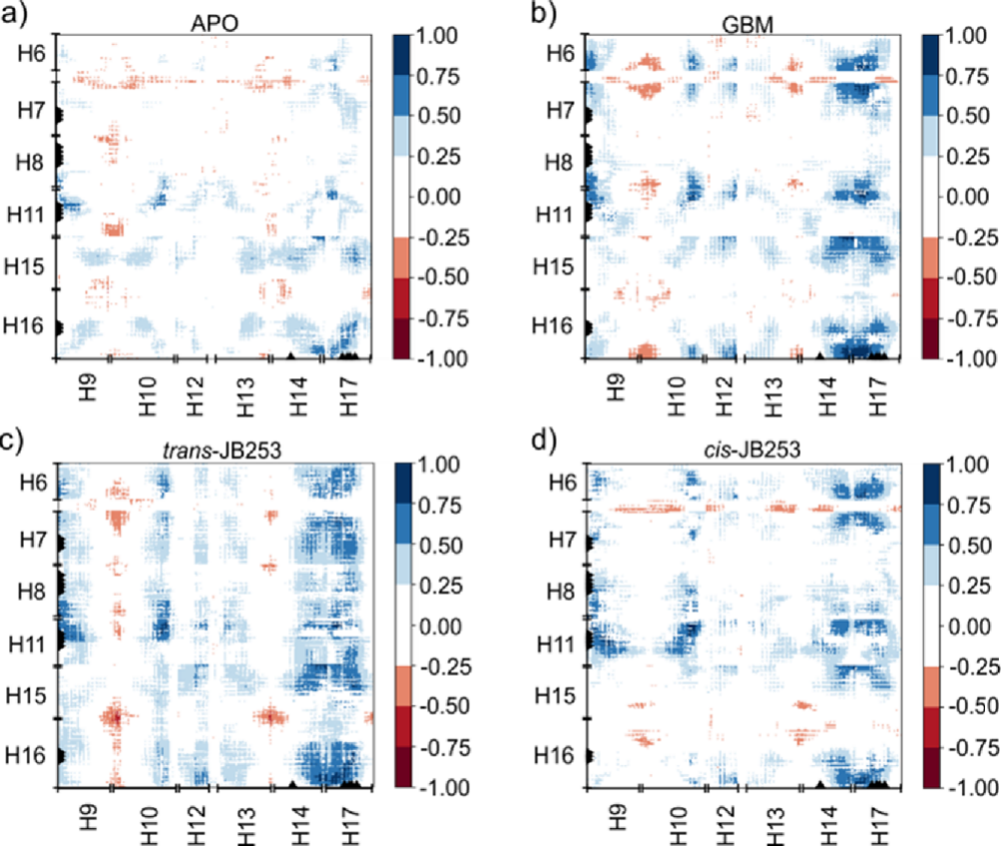
Correlation matrices calculated from 5 × 200 ns MD trajectories data for (a) APO, (b) GBM-SUR1, (c) *trans*-JB253-SUR1, and (d) *cis*-JB253-SUR1 systems. The horizontal axis corresponds to L2 (helices H9, H10, H12, H13, H14, and H17), while the vertical axis corresponds to helices forming L1 (H6, H7, H8, H11, H15, and H16).

Couplings among parts of the SUR1 protein (see Figure 2) were analyzed using the dynamic cross-correlation (DCC) matrices.^46^ It is a measure of similarity of two amino acid residues as a function of their relative displacement throughout the trajectory. We calculated the residue–residue contact score^47^ (RRCS) for each drug in each frame to determine which residues in potential binding sites interact with the drug. RRCS quantifies a statistical frequency of a ligand–residue contact by summing all possible contributions from heavy-atom pairs. RRCS is defined as
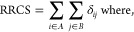

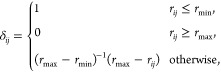
where *r_ij_* is the distance between the *i-* and *j*-th atom and *r*_min_ = 3.23 Å and *r*_max_ = 4.63 Å. In our case, group A contains atoms of the ligand and group B consists atoms of the whole protein.

For 3D visualization of molecular systems, we used VMD.^48^ The charts and plots were prepared using the Matplotlib Python package.^49^

## 3 Results and Discussion

### 3.1 Computational Models (APO, GBM, *cis-*JB253, and *trans-*JB253)

The structure of the KATP channel, despite its physiological role, remained unknown for a long time. In 2005, the first cryo-EM data on the rat-mouse construct of Kir6.2/SUR1 has been reported,^50^ but the resolution was low (18 Å). In 2017, higher resolution (<4 Å) cryo-EM structures were published.^24,25,27,28^ SU drugs bind easier to the open form of SUR1 due to a better access route. Unfortunately, the experimental structure of human SUR1 in this open conformation has not been published yet. Therefore, the open form required for this study was prepared using the targeted MD method^30^ (see the Methods section). We focus the modeling on a single SUR1 domain embedded in a phospholipid membrane slab, with neither ATP nor ADP ligands in nucleotide-binding domain (NBD) pockets.

In this project, we investigated changes in protein dynamics induced by three ligands. The first two were the aforementioned cis and trans forms of JB253 (*cis*-JB253 and *trans*-JB253), 1-cyclohexyl-3-[4-[[4-(diethylamino)phenyl]diazenyl]phenyl] sulfonylurea (CCDC: 1014606). The third ligand, serving as a reference, was the prescription drug glibenclamide (CAS: 10238-21-8, GBM). In Figure 1b, we show three functional groups U (cyclohexyl and urea groups), S (the sulfonyl group), and R (the remaining part) of all ligands. R is a phenyl ring linked to the chloro benzamidoethyl group for GBM and the azobenzene group for JB253. The light-induced isomerization involves a decrease in the distance between the two carbon atoms in position 4 of the aromatic rings of azobenzene, from 9.0 Å in the trans form to 5.5 Å in the cis form.^51^ The distances between the cyclohexane and phenyl moieties are 13.25, 13.83, and 8.39 Å for GBM, *trans*-JB252, and *cis-*JB253, respectively, so *cis-*JB253 is the most compact and bent.

One should note that the binding affinity of GBM to SUR1 protein is almost 1000-fold higher than that of JB253 (IC50 = 4.2 nM vs 17.6 μM for *trans*-JB252, see Table S1). There is also a qualitative agreement between relative IC50 values and docking scores obtained in our modeling. In simulations, we always consider a single ligand in one protein pocket, and thus concentration effects were neglected.

The ligands were docked into the open SUR1 structure using Glide 2018.4.^34^ Every ligand is lodged in the transmembrane bundle of the SUR1 ABC core near the inner leaflet of the lipid bilayer (Figure 1a,c; a dashed oval). All SUs are encompassed by a similar set of residues from helices H7, H8, H10, H16, and H17 (SI, Figure S2). Our docked structure of GBM overlaps with the binding site reported from the cryo-EM experiment. For the APO protein and all ligand-SUR1 systems, we ran five repeats of unbiased MD simulations with a total length of 1 μs for each model.

### 3.2 Ligands Affect Unbiased Molecular Dynamics of SUR1

SUR1 has a structure (Figure 1) typical for ABC transporters (ABCC8). Two intracellular nucleotide binding domains (NBD1 and NBD2) are attached to bundles of helices forming shafts, called “legs” L1 (proximal to Kir6.2 subunit of KATP) and L2 in this paper. We prefer using these names instead of standard TMD1 and TMD2 since those two legs exhibit a domain swapping between TMD1 and TMD2: H15 and H16 from TMD2 belong to L1 and H9, and H10 from TMD1 belong to L2. Cryo-EM and other experiments^29^ show that upon magnesium-adenosine-diphosphate (MgADP) binding to NBD2 and NDB1, both domains stack together, and SUR1 adopts the closed form (outward open). When just one adenosine-triphosphate (ATP) molecule replaces ADP, which happens due to an elevated level of ATP produced by mitochondria after glucose intake, the domains NDB1 and NDB2 dissociate and move apart, and the open SUR1 form (inward open) is observed (see Figure 1a).

Using five unbiased, 200 ns long, MD trajectories for APO SUR1 and each ligand docked in the SU pocket, we calculated correlation matrices (see Methods for a definition) for the transmembrane helices of our model (Figure 2). Maximum errors in correlation matrices data are presented in Figure S4 (SI). In Figure 2, helices belonging to L1 keeping the NBD1 domain (H6, H7, H8, H11, H15, and H16) are represented on the *Y*-axis, and those comprising the other L2 leg on the *X*-axis (H9, H10, H12, H13, H14, and H17). Negative values (shown in red in Figure 2) indicate the anti-correlated motions of the appropriate helices.

Analysis of correlation matrices shows that SU ligands affect unbiased molecular dynamics of SUR1. For possible modulation of the KATP channel state, the most interesting are correlations (L2 and L1) between L1 and L2 fragments described by (H9-H10, H15-H16) and (H13-H14, H15-H16) regions. Data in Figure 2 show that in all systems, there are red regions. These large negative values denote that the corresponding helices are moving in the opposite directions. Thus, scissor-like closing–opening motions of SUR1 L1 and L2 parts are mainly observed in those parts. Based on our limited statistics (only 4000 frames from total 1 μs trajectories were analyzed, see Figure S4 for analysis of errors), it is difficult to measure the impact of drugs on the dynamics of those regions, but the strongest anti-correlation is observed for the *cis*-JB253-SUR1 system (Figure 2d).

Another effect of the ligands’ presence in SUR1 is seen in the region H14-H17 (L2), which clearly exhibits increased correlation with many regions of L1 after ligand binding. The increase in H14-H17 correlation stems from the stabilization of L2 in the SU binding pocket region. Here, a long *trans*-JB253 molecule has the largest impact on SUR1 dynamics (cf. Figure 2a,c).

The detailed mechanism of the sulfonylurea action on the KATP channel despite many efforts^28,52,53^ is not known yet. From structural studies, we know that the presence of the drug inhibits the dimerization of NBD domains and possibly facilitates the anchoring of the N-terminal part of Kir6.2 (KNt, for more details, see Figure S1) into the SUR1 cavity.^33,52,53^ The KNt fits well into the SU cavity, and it was detected in that region in the presence of the GBM drug.^28,53^ Both SU drugs and the presence of N-terminal promote an open form of SUR1, and that form increases the probability of KATP channel closure. Two issues, poorly resolved in cryo-EM studies, are particularly important: (1) to what extent does the presence of a ligand lead to such significant conformational changes of SUR1 and (2) how that conformational signal is further transmitted to the Kir6.2 pore in the KATP channel?

**Figure 3:**
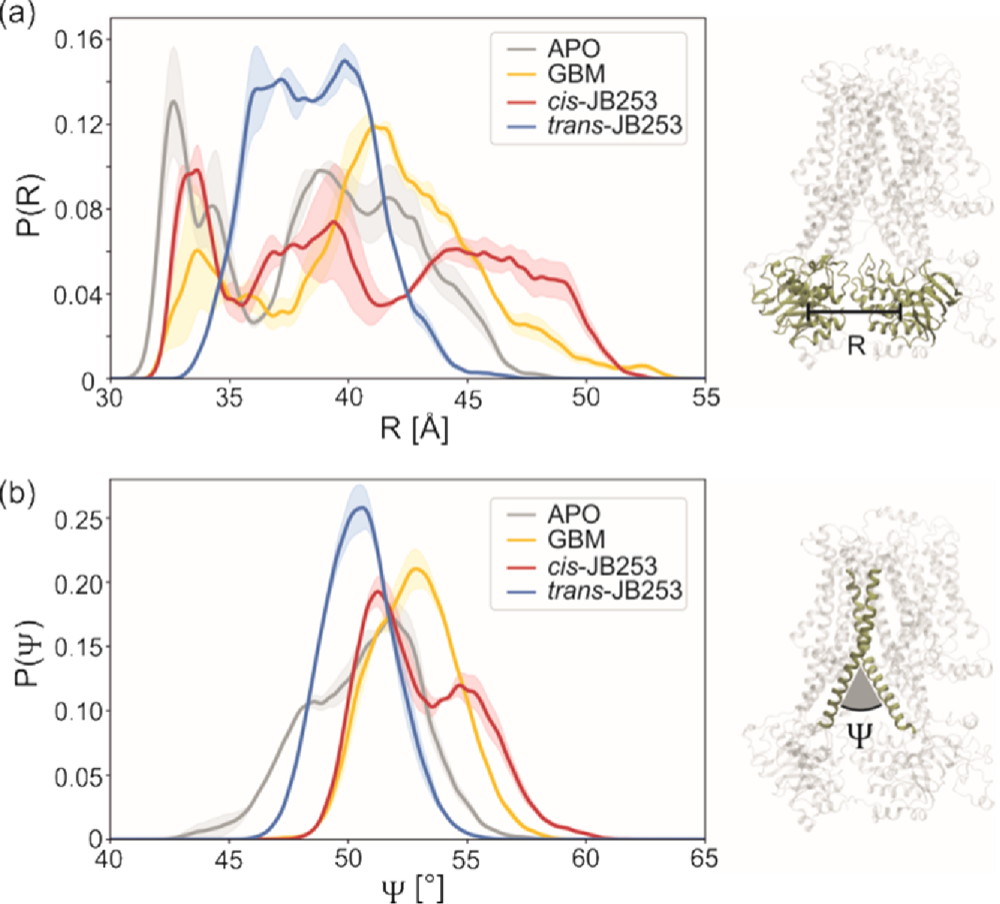
Distributions of NBD1-NBD2 distances *R* (a) and angles Ψ between two selected transmembrane helices H9 (L2) and H15 (L1) (b) calculated from 1 μs MD simulations. The probability density functions are normalized. Estimated errors are indicated by shadow areas.

**Figure 4:**
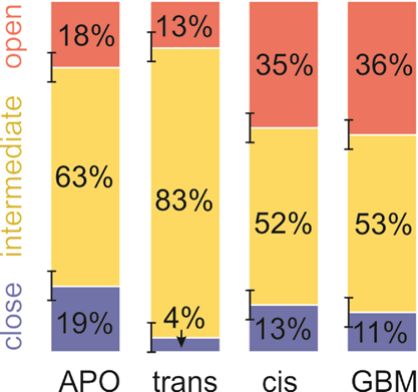
Populations (in percentage) of different conformational states of SUR1 (APO) and SUR1 with SUs docked with JB253 (trans and cis) and GBM in total 1 μs MD simulations. Estimated errors are indicated by vertical black bars.

Since our model system consists of a single SUR1 part of the KATP channel alone, we can address only the first issue. To monitor conformational changes in SUR1 dynamics, we defined two geometrical parameters: (1) the distance *R* between centers of mass of the NBD1 and NBD2 domains and (2) the angle Ψ sensitive to the L1 part bending. In Figure 3a, distributions of *R* distances in all systems observed during MD simulations are shown. Clearly, in all cases, at least binominal distributions are present. To facilitate analysis, we classify all conformers with *R* < 35.0 Å as closed, 35.0 Å < *R* < 42.0 Å as intermediate, and *R* > 42.0 Å as open SUR1 forms (see Figure 4). Despite the absence of nucleotides in our NBD domains, which may lock SUR1 in the closed form, the calculated difference between *R* in the arbitrarily chosen open and closed states (Δ*R*) of 7 Å compares rather well with an experimental shift Δ*R* of 11 Å observed in hamster SUR1 structures (6BAA: open,^25^ 5YWC: closed^28^). The most striking observation is a clear shift of the GBM distribution toward open structures. In contrast to that, the distribution of *trans*-JB253 conformations favors the intermediate structures and has a minor component of the open structures. The photo-excitation of JB253, leading to trans → cis transition, modifies the calculated distribution dramatically (Figures 3a and 4) and makes it much wider and more similar to GBM. Structures with large values of *R* (i.e., the 45–47 A region, Figure 3a) are observed for *cis*-JB253 data as well. The effects of ligands on the frequency of open and closed forms of SUR1 are summarized in Figure 4. Note that populations of *cis*-JB253 and GBM drugs are almost identical. Notably, checking the convergence of our simulations, we estimated that the error in determination of percentage of close/intermediate/open conformations (Figure 4) does not exceed 4%. The errors indicated in Figure 3 and in Figure 4 were determined by collecting the maximum deviations of a given parameter (R, Ψ, and %) calculated for an increasing length of trajectories from 150 to 200 ns (in 10 ns steps).

We infer from the threefold increase in open conformation probability occurrence (red bars in Figure 4) that photo-isomerization of JB253 shifts the equilibrium established for the ground state *trans*-JB253 bound to the protein substantially and makes SUR1 more prone to adopt the open form. As we know, opening of SUR1 correlates with a higher probability of Kir6.2 pore closing.^16,54^ The closed Kir6.2, and thus the closed whole KATP channel, affects the β-cell membrane potential.^54^ Therefore, in that way, the light-induced conformational change of JB253 may affect insulin release from β-cells, as observed by Broichhagen et al. in 2014.

The more open character of both GBM and *cis-*JB253 SUR1 structures is further supported by distributions of the Ψ angle between L1 and L2 parts (see Figure 3b). All plots have a binominal character again and are both similarly shifted toward larger values of the “scissors” Ψ angle. The maximum probability densities P(Ψ) of 52, 53, 50, and 51° are observed in APO, GBM, and *trans-* and *cis*-JB253, respectively. Calculated values should be compared to Ψ = 48° in the closed and 56° in the open form of hamster SUR1.^25,28^

To conclude this part of the MD analysis, we want to stress that water molecules have easy access to the SU cavity. In the SI, we present data (Figure S5 and Table S3) showing that closed and open forms of SUR1 accommodate different numbers of water molecules. In a somewhat arbitrary selected sampling region, after averaging over appropriate subsets, we observed 297 (sd. 18) water molecules for the open and 244 (sd. 19) for the close form of APO SUR1. While the ligands are present, the number of water molecules decreases by ca. 15 molecules. The volume occupied by water is large enough that the sampled region in a real KATP channel is likely occupied by the KNt tail of Kir6.2, a polypeptide probably involved in the allosteric regulation.^33^

### 3.3 Metadynamics Confirms the Light-Induced Opening of SUR1

**Figure 5:**
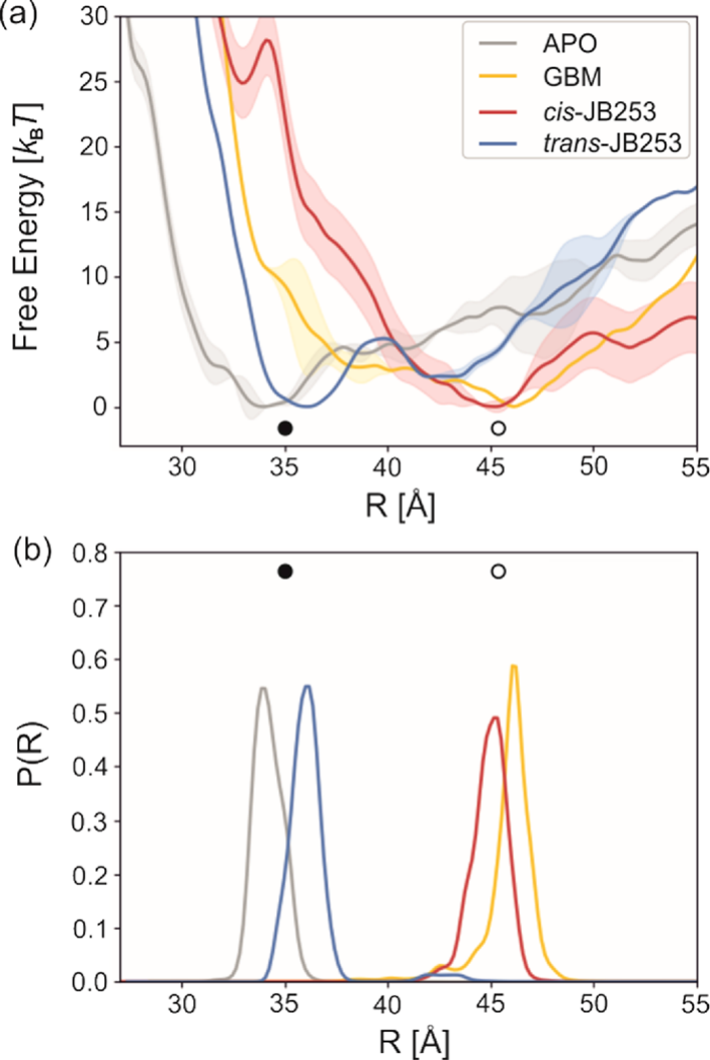
Free energy profiles for close (solid circles) to open (open circles) transitions in SUR1. *R* is the distance between centers of mass (COM) of NBD1 and NBD2 domains (a). Distributions of probabilities for finding SUR structures of the given NBD1-NBD2 distance (b). The probability density function *P*(*R*) in each case is normalized. Errors of free energy profiles (convergence) are shown as the largest deviation of 130 ns and consecutive profiles from the presented 170 ns plots.

In the unbiased 1 μs MD simulations, a wide range of APO SUR1 conformations was observed, as indicated by the distribution of *R* distance between NBD1 and NBD2. We used metadynamics (see Methods) to calculate the free energy (Δ*F*) profiles along this coordinate. Based on the unbiased trajectory analysis, we assume that this coordinate describes a transition between open and close structures of SUR1. The results are presented in Figure 5. For the APO form, despite starting metadynamics simulations from an open conformation, the Δ*F* profile shows a minimum at *R* = 34 Å, which we classify as a closed form (Figure 5).

There is no profound local minimum for the open form. The calculated energies of APO open are some 5 *k*_B_*T* (3 kcal/mol) higher than the close one. The binding of GBM reverses this situation: the most stable conformation is the open one (*R* = 46 Å), and energies of the closed conformations are 4 *k*_B_*T* (2.5 kcal/mol) higher. JB253, docked in its trans conformation, promotes the closed conformation of SUR1 (*R* = 36 Å), similar to APO. In contrast, the *cis-*JB253 geometry, mimicking the structure and charge distribution achieved via a photo-excited state, drives SUR1 into an open form (*R* = 45 Å). Thus, metadynamics shows that photo-excitation results in a shift of the NBD1-NBD2 preferred distance by 9–10 Å. This phenomenon explains the molecular mechanism of experiments performed by Broichhagen et al.^10^ The open form of SUR1 leads to a more frequent closing of the KATP channel, depolarization of the β-cell membrane, calcium influx, and enhanced insulin release.

### 3.4 Local Interactions of Sulfonylurea Ligands in the SUR1 Pocket Allow Signaling to Kir6.2 Units by Their N-Terminal Stabilization

**Figure 6:**
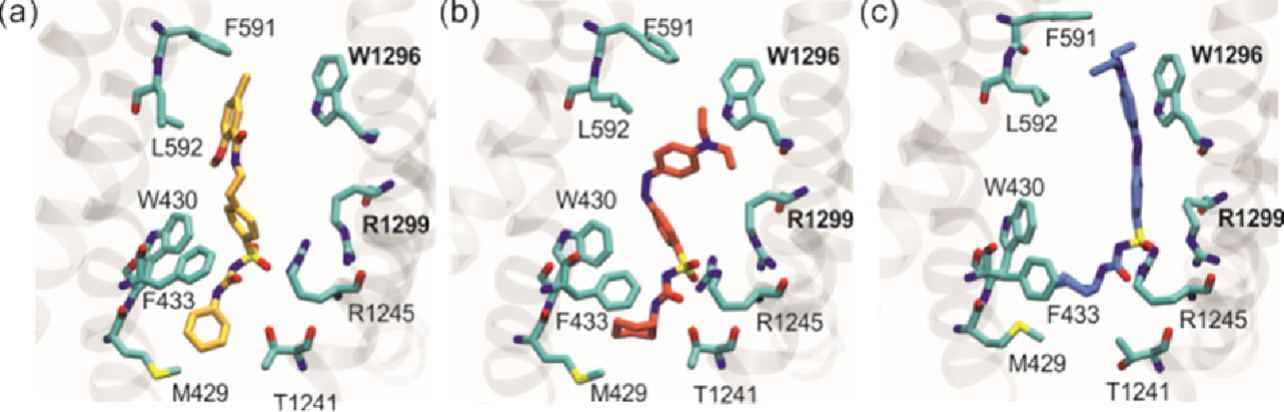
Representative snapshots of the drug and its surroundings for each system: GBM (a), *cis*-JB253 (b), and *trans*-JB253 (c).

According to the cryo-EM data,^23,25,26,33,34^ the SU binding pocket in SUR1, shown in Figure 6, is located between L1 and L2 legs, in the “hinge” region. It is delineated by the “upper parts” of H7, H8, H11, H16 (from L1), and H17 (from L2) helices. The estimated volume of this cavity, based on the 6PZA PDB structure, is 3000 Å^3^. That large volume is accessible to N-terminus (KNt, aa 1–31) from Kir6.2, provided that SUR1 is in the open conformation.^34^ Due to its inherent flexibility, the position of KNt is poorly resolved in cryo-EM structures, but it has perhaps a major regulatory role in the control of KATP channel closing.^26,30,33,34^

Under physiological conditions, there is an equilibrium between the closed and open forms of SUR1.^16,55^ According to the model proposed by Wu et al.,^28^ in response to an increase in the ATP/ADP ratio occurring after glucose uptake to the β-cell, the ligation state of NBD domains changes. The interaction between NBDs becomes weaker, which results in SUR1 adopting an open form more often. An increased volume between the L1 and L2 parts of SUR1 units facilitates penetration of cavities and KNt wedges into the SU pocket. That event blocks the scissor motions of SUR1. Thus, the steric hindrance generated by the presence of KNt in the SU cavity results in an increased probability of the open conformation occurrence. The strained KNt locked in SUR1 relays a signal to the central (Kir6.2) part of the channel. This leads, in turn, to an increased probability of Kir6.2 pore closing. Our docking of SU ligands and MD simulations show that the preferred sites of GBM and JB253 are in the SU region. However, the dynamical behavior of ligands is different—the *trans*-JB253 is relatively stable through all generated trajectories (in total 1 μs sampling time), while both GBM and *cis*-JB253, due to less stabilizing interactions with the protein and their conformational flexibility, promote SUR1 opening.

#### 3.4.1 Positions of Docked Ligands in the SU Cavity Are Similar

**Figure 7:**
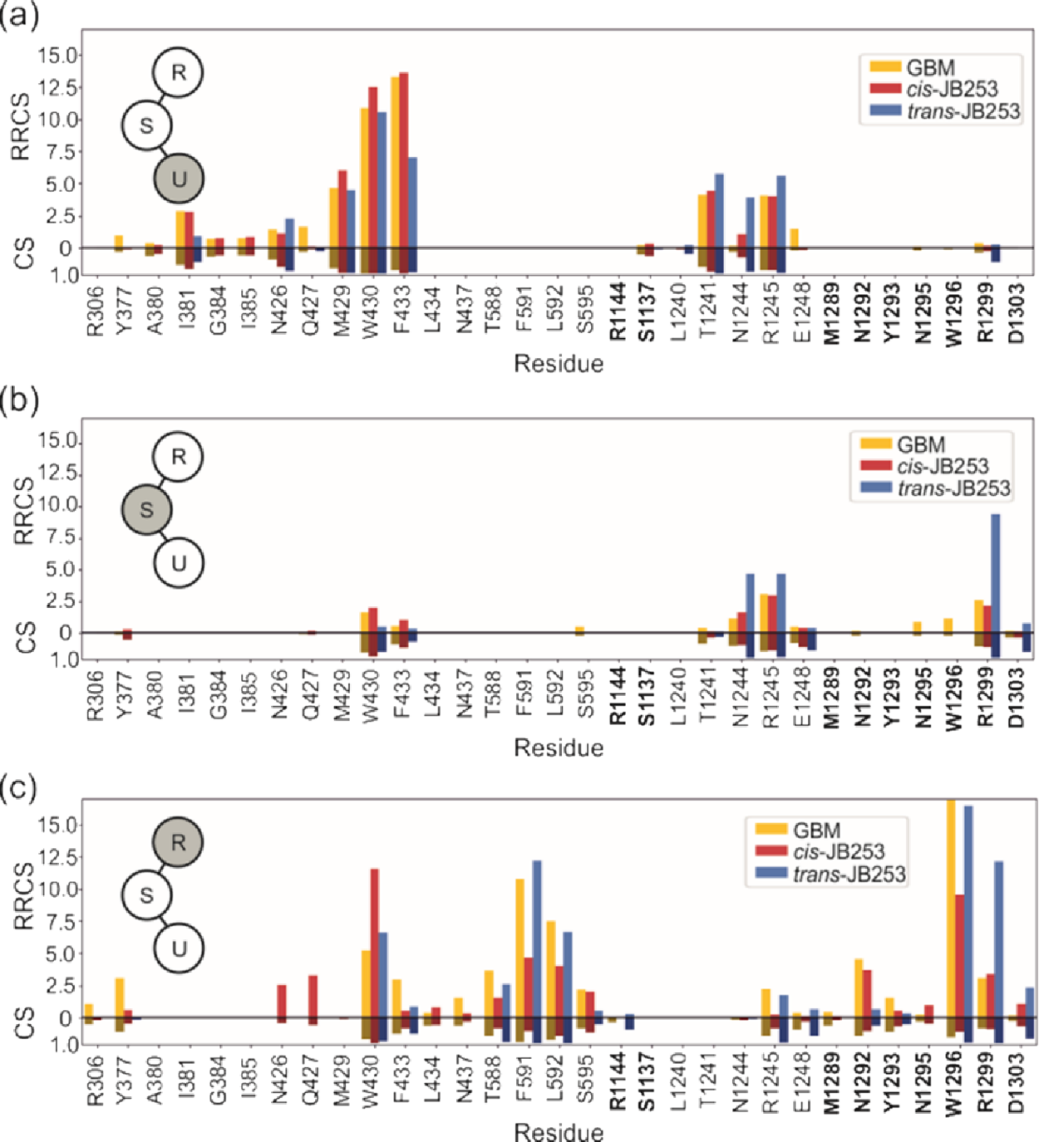
Residue–residue contact score (RRCS) and a standard contact score (CS) between protein residues and respective parts of ligands: urea and cyclohexyl group – U (a), sulfonyl group – S (b), and the remaining part – R (c), which is the azobenzene group in JB253 and the chlorobenzamidoethyl group in GBM. Residues belonging to L2 are indicated in bold.

**Figure 8:**
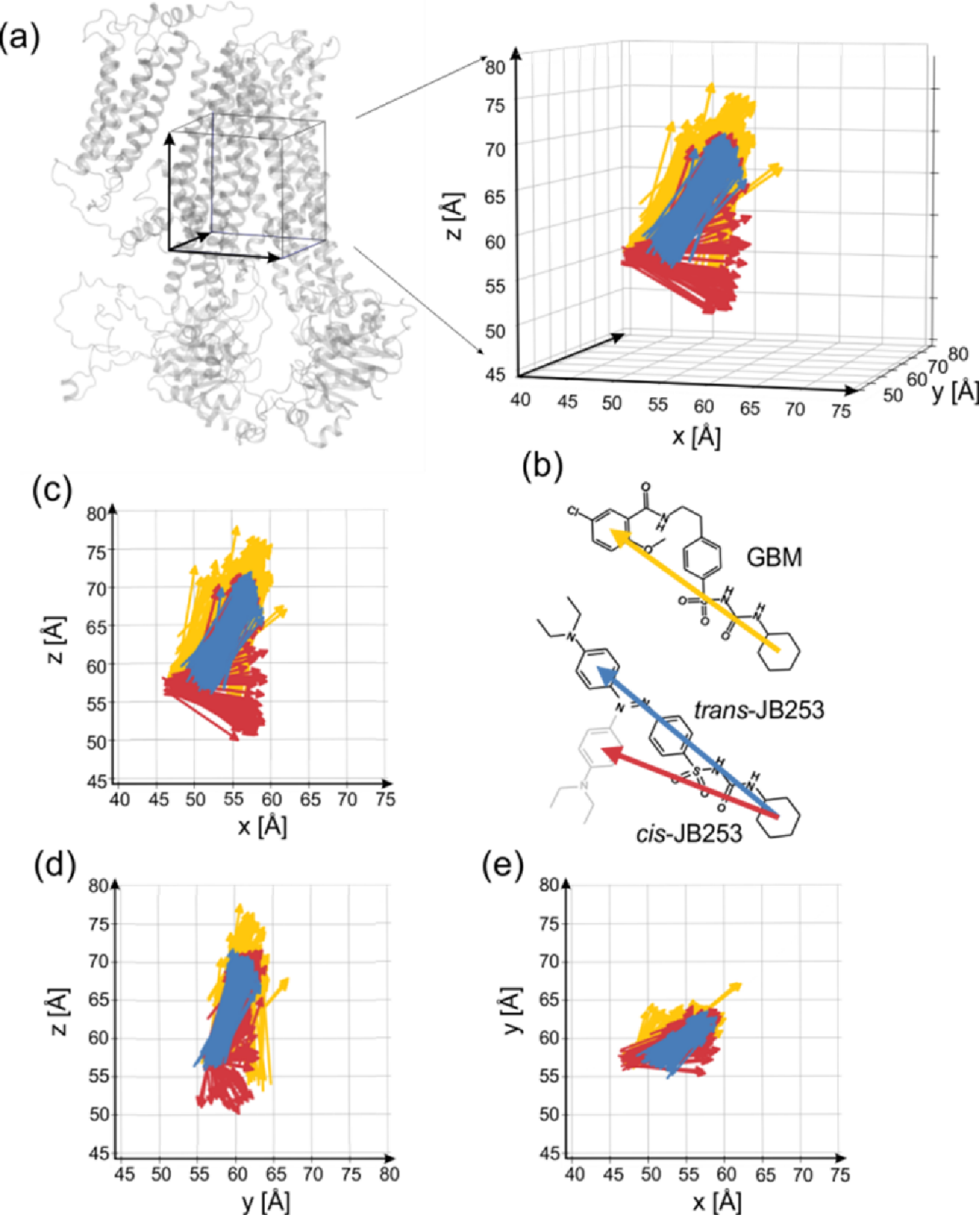
Distributions of ligands orientation extracted from 1 μs trajectories. *Z* axis corresponds to the long axis of SUR1, and the orientation of the molecule is shown in Figure 1, negative *x* – L1 and positive *x* – L2. (a) Arrows link the center of mass (COM) of the cyclohexyl group and COM of the “upper ring” for yellow – GBM, blue – *trans*-JB253, red – *cis*-JB253 (b). Three projections of ligands’ orientations (c–e).

According to cryo-EM data,^52,53,56,57^ GBM binds in the SU pocket very closely to KNt, being in contact with AA2, AA4, and AA5 from KNt. The docked pose of GBM (Figure S2a) is in very good agreement with the cryo-EM one.^53^ The sulfonyl group of GBM has interactions with two arginine residues belonging to L1 and L2 (Arg1245:H16:L1 and Arg1299:H17:L2). Notably, mutation of those residues completely abolishes the inhibition of KATP by both sulfonylureas and glinides.^57^ The urea moiety (see Figure 1) interacts with Thr1241 and Asn1244. Met429 and Trp430 stabilize this moiety as well. The “lower” part of the SU pocket is delimited by Ser1237—the cyclohexane ring is located in its vicinity. Due to its large size, the unique part of GBM, namely, the chlorobenzamidoethyl group (see Figure 1), is important for the nanomolar binding affinity of this antidiabetic drug. That group is encircled by a ring of hydrophobic side chains: Y377, W1296, and L592, which accommodates the benzene ring of GBM, while N437 and R306 stabilize the ligand through the Cl and methoxy group. Notably, those two interactions are, to a large extent, lost during MD simulations (see Figure 7a). In all (5 × 200 ns) MD trajectories of the GBM-SUR1 system, the GBM drug remains roughly in the original docking position in the SU pocket. However, it exhibits some conformational freedom (RMSF = 5 Å), which is expected, given the absence of KNt in our computational model (see Figure 8 and SI, Figure S6).

Both forms of JB253 were docked in the same region of the SU pocket as GBM. The sulfonyl group of JB253 is present in the region of Arg1245 and Arg1299, but arginines’ hydrogen bond donors are linked to the urea group atoms as acceptors (Figure 6b,c and SI, Figure S2) instead of sulfonyl oxygens. The JB253 urea moiety (including the cyclohexane ring) has the same partners as in GBM: a ring of Ser1237, Thr1241, Asn1244, and W430 plus hydrophobic Leu1240 and Ile381. The unique for the JB253 light-sensitive N-(dietyl)-azobenzene group, we named the R group (see Figure 1). As mentioned, the trans conformer is 4 Å longer than the cis one. This minute change in the molecular shape, induced by absorption of 460 nm photons, modulates insulin release from β-cells.^10^ Our docking does indicate that the R group in *trans*-JB253 protrude deep into a bay formed by hydrophobic Leu592, Phe591, Trp1296, and Val587 and hydrophilic Thr588, Asn547, and Arg1144 residues. In contrast, the bent R group of *cis*-JB253 retains contacts with the first three hydrophobic residues only (SI, Figure S2). Therefore, *cis*-JB253 is less stabilized than *trans*-JB253.

#### 3.4.2 Dynamics of Sulfonylurea Ligands in SU Cavity Is Different

Sulfonylurea compounds studied here exhibit different conformational behaviors on a sub-μs time scale. To check their dynamics, we monitored residue–residue contact scores (RRCS) and classical contacts (CS) and averaged data over all frames. The results are presented in Figure 7. Here, we wanted to extract possible differences in the dynamics of GBM and JB253 in trans and cis forms. The same sets of SUR1 protein residues interacting with the urea groups (U) of all ligands (M429, W430, F433; T1241, R1245) is observed (see Figure 7a). The values of RRCS and CS for GBM are similar, but those for *trans*-JB253 are lower for M429, W430, and F433 and higher for N1244 and R1245. In the S region, there are more profound differences between cis and trans forms of JB253: R1245 and R1299 are much often in contact with trans than with the *cis*-JB253 conformers (Figure 7b).

Particularly striking is the difference between the high *trans*-JB253 RRCS value for R1299 and low values for GBM and cis structures. Both R1245 and R1299 stabilize the ligand by interacting with the sulfo group oxygens, but in the case of *trans*-JB253, stabilization is better. One can hypothesize that keeping those L1 and L2 contacts short for an extended time stabilizes the closed conformation of SUR1. Therefore, *trans-*JB253, being relatively stable in MD (see Figure S6), does not allow for opening fluctuation that might facilitate entering the NKt tail to the SU pocket. In contrast to that, both GBM and *cis*-JB253 lose contact with R1299 and facilitate a wider opening of the L1-L2 cleft. Spatial distributions of vectors representing orientations of GBM and JB253 ligands clearly illustrate the strong localization of *trans*-JB253 and elevated conformational fluctuations of GBM and yet much higher conformational freedom of *cis-*JB253 (Figure 8). The stronger localization of *trans*-JB253, with respect to the other ligands studied, results from a good fit of the *trans*-azobenzene moiety between aromatic rings of W1296 and F591. A deeper analysis of residue positions crucial for protein–ligand interactions, shown in tomograms presented in Figures S8 and S9 in the SI, further supports this observation. Therefore, we infer that KNt may be trapped in the SU pocket with a higher probability when GBM or *cis-*JB253 is present in its vicinity. Dynamical modeling data clearly show that GBM-SUR1 and *cis*-JB253-SUR1 are more prone to adopt the open conformation.

#### 3.4.3 Biological Significance

It is plausible to postulate that both GBM and *cis*-JB253 ligands stabilize the physiologically important allosteric KNt tail better than the localized and rigid *trans*-JB253 or SUR1 without any secretagogue attached to the SU pocket. This model explains the molecular basis of the SU antidiabetic drug activity related to interactions with KATP pancreatic channels. To what extent do the expected interactions between the ligand and the KNt stabilize a strained position of the Kir 6.2 N-tail is a matter of separate modeling.

Our study addresses a general problem of allosteric control of an ion channel function. Physical factors triggering conformational changes, such as interactions with agonist or antagonist, are central for regulating the metabolism. KATP channels involved in a precise regulation of the blood glucose level are quite old in terms of evolution they are present, i.e., in fishes.^58^ The system studied here is a paramount example of remote chemical signal transduction: a state of one protein (conducting/non-conducting Kir6.2) is affected by a conformational change (close/open) of another associated protein (SUR1). Both cryo-EM data^28,53^ and our modeling show that the major route of signaling initiated by a protein sensor reporting the ATP/ADP ratio (SUR1) is by a 20 Å long unstructured tail of another partner. Such a mechanism of allostery, based on docking of a disordered polypeptide fragment, is expected to play a role in other protein complexes as well.

Proteins associated with ion channels are quite common, i.e., in the voltage-gated calcium channel (Ca_V_) pore-forming subunit α-interaction domain (AID) and cytoplasmic β-subunit (Ca_V_β) are coupled through a peptide part of Ca_V_ docked to a groove in Ca_V_β.^59^ Auxiliary subunits in BK potassium channels that regulate the firing of neurons and neurotransmitter release are, similar to our KATP system, linked to the conductance pore by disordered protein fragments.^60^ Such disordered peptides are also critical in plant cryptochromes where light-induced dimerization leads to binding of regulatory proteins.^61^ Experiments with photo-activated sulfonylureas indicate that the protein function might be controlled by exogenous ligands, and we have delineated molecular basis of such control.

We determined a set of the SUR1 residues involved in a signal transduction pathway in the SUR1 part of KATP. This opens the possibility of rational mutational studies, especially with the usage of photo-activated ligands. Since localized light-induced conformational changes shift the open/close equilibrium in this type of ABCC8 transporter, similar physical stimuli might be introduced to other complexes as well. Thus, due to carefully designed azobenzene-based ligand molecules, a new biophysical research area of “protein photo-mechanics” may be envisaged. The success of optogenetics^2^ should be transferred to other areas of molecular biology as well.^62^

## 4 Conclusions

In this work, we present a step toward elucidating a mechanism of drug-induced insulin release from pancreatic β-cells. The high metabolism state renders binding of ATP to inhibitory sites in Kir6.2 and MgATP to the NDB domains of SUR1 and results in KATP channel closing.

Using MD simulations of the SUR1 model, we determined conformational dynamics and local interactions of antidiabetic drug glibenclamide localized in the central SU pocket. We confirmed that both trans and cis conformers of light-sensitive sulfonylurea JB253 dock to the same cavity. Residues Arg1245, Arg1299, and Trp430 encompass GBM or JB253 ligands. We identified major structural changes in SUR1 correlated with the trans and cis conformers of JB253 and showed that *cis*-JB253 has features closely resembling the GBM-SUR1 structure. *Cis*-JB253 is more prone to promote an open SUR1 than the conformer trans. In its open form, SUR1 has an elevated chance to accommodate the N-tail of Kir6.2 in the central SU cavity. Similar to Martin et al.,^53^ based on dynamical data, we postulate that the presence of GBM or *cis*-JB253 further stabilizes this coupling. The N-tail relays signals from SUR1 to the Kir6.2 part of the KATP channel, and it is indispensable for the proper functioning of this molecular switching machine.^28,53^

Simulations show that light-induced conformational change in JB253 sulfonylurea opens more space, enabling more effective and deeper KNt docking/wedging, and this perhaps leads, in turn, to the KATP channel closing. Furthermore, calculated free energy profiles along the NBD1-NBD2 distance show that *trans*-JB253-SUR1 prefers the closed conformation, but both GBM and *cis*-JB253 favor the open one. Therefore, we computationally confirmed and explained, at a molecular level detail, the results of experiments demonstrating light-controlled insulin release in pancreatic β-cells.^10^

The present study opens a way to further improve photoactive drugs.^63^ Based on SUR cavity dynamics and architecture, new functional groups in part R may be proposed, increasing a relatively weak JB253 binding affinity toward the channel. Notably, SUR proteins, as partners in KATP channels, are present also in cardiomyocytes^22,64^ and are involved in neuronal tissue injuries;^65^ therefore, prospects for new medical applications of photo-pharmacology are bright.^11^ In particular, molecular details of drug-SUR1 interactions should help to reduce possible adverse effects of sulfonylureas by reducing unwanted binding to cardiac SUR2 proteins. Moreover, polymorphisms in SUR genes, sometimes linked with genetic diseases, may be correlated with the role of critical residues delineated in this work.

## Supporting Information Available

Further computational details and data (PDF)

## Notes

Structures of ligands, informative snapshots from trajectories, and all inputs for MD and metadynamics simulations are deposited in Github https://github.com/kszewc/KATP and in the PLUMED Consortium repository under plumID:21.031. Any other information needed to reproduce our results are available from authors upon request.

## Abbreviations

KATP: ATP-sensitive potassium channel
SU: sulfonylurea
GBM: glibenclamide
Kir6.2: inward rectifying potassium channel
SUR1: sulfonylurea receptor type 1
KNt: N-terminal part of Kir6.2
T2DM: type II diabetes
TMD: transmembrane domain
NBD: nucleotide binding domain
cryo-EM: cryogenic electron microscopy
MD: molecular dynamics
JB253: 1-cyclohexyl-3-[4-[[4-(diethylamino)phenyl]diazenyl]phenyl] sulfonylurea
ATP: adenosine triphosphate
ADP: adenosine diphosphate
PDB: Protein Data Bank
COM: center of mass
RRCS: residue–residue contact score
CS: contact score
WTM: well-tempered metadynamics
