# Supplementary material for: Photo-Switchable Sulfonylureas Binding to ATP-Sensitive Potassium Channel Reveal the Mechanism of Light-Controlled Insulin Release
Source: J Phys Chem B. 2021 Nov 26;125(48):13111–21. doi: 10.1021/acs.jpcb.1c07292 (PMC8667036; doi:10.1021/acs.jpcb.1c07292)
Supplement: Supplementary file 1 — jp1c07292_si_001.pdf [file jp1c07292_si_001.pdf]

Supporting Information for Publication

Photo-switchable Sulfonylureas Binding to ATP-sensitive Potassium Channel Reveals Mechanism of Light-controlled Insulin Release

Katarzyna Walczewska-Szewc, Wiesław Nowak*

Faculty of Physics, Astronomy and Informatics, Nicolaus Copernicus University in Toruń, 87-100 Toruń, Poland

*wiesiek@fizyka.umk.pl

KATP channel

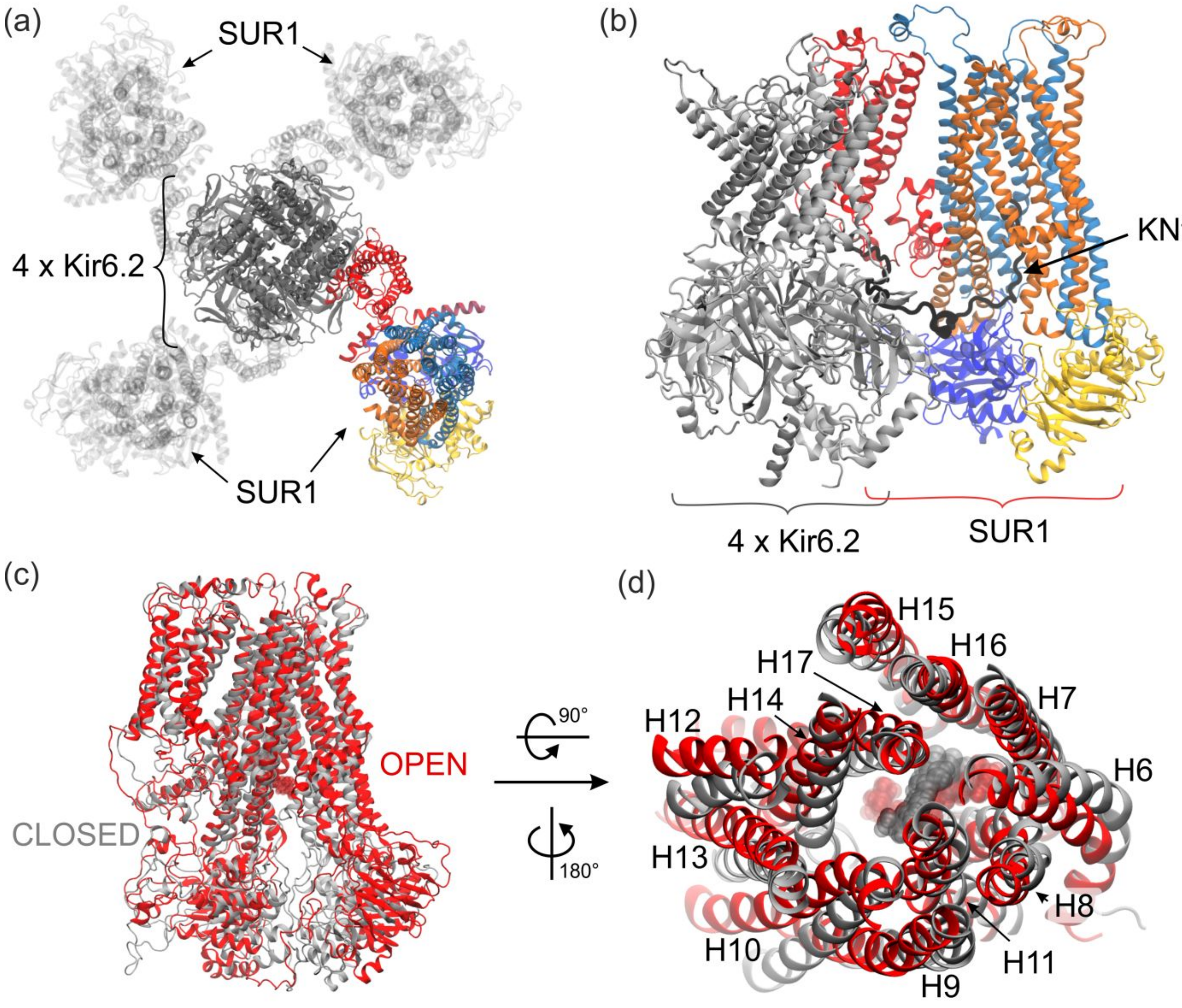

Figure S1. A general scheme of the KATP channel architecture (PDB ID: 6C3P) – top (a) and a side partial view (b). The pore transmitting potassium ions outwards (from bottom to top, Fig. 1 b) is formed by four Kir6.2 subunits. The closing/opening of this pore is related to ATP/ADP ratio and is perhaps regulated (i.a.) by the N-terminal tails (KNt – black) of each Kir6.2 protruding into the SUR1 helper protein. Overlay of open (red) and closed (gray) structures of SUR1 (c). Rearrangement of helices in TMD1 and TMD2 domains in open (red) and closed (gray) states (d). Cis and trans forms of JB253 are shown as red and gray transparent volumes.

Preparation of ligands parameters

The 3D structure of GBM was extracted from CryoEM structure PDB code 6BAA. Structure of *trans*-JB253 was downloaded from the Pubchem database (CID: 78060683). As a first approximation of the cis state of JB253, the C- N=N-C dihedral angle was changed manually to 180°. The geometry of all ligands was then optimized using GAUSSIAN (the DFT method B3LYP 6-311**). Due to interactions with asymmetrically linked cyclohexane moiety, the chiral conformer P of *cis*-JB253 was energetically favoured. CHARMM36 force field parameters were obtained from the Swissparam server (www.swissparam.ch), using the optimised ligand structures as an input.

To validate the parameters of the drugs, we calculated the octanol-water partitioning coefficients, which were compared with available experimental data. The octanol-water partitioning free energies were determined using the Bennett Acceptance Ratio (BAR) approach implemented in MDPOW- a python package that automates the calculation of solvation free energies via molecular dynamics (MD) simulations and facilitates the computation of partition coefficients (https://github.com/Becksteinlab/mdpow).

First, GBM and trans-JB253 were solvated (in water or the water-octanol mix) in a periodic dodecahedron simulation cell with a minimal distance of 2 nm to the nearest box surface. The NPT ensemble MD simulations were performed at T=3oo K for 20 ns, with all other parameters kept as MDPOW default (for CHARMM force-field simulation)[1]. Simulation parameters for water and octanol simulations were identical. Solvated systems were first energy minimized and relaxed with a short, five picoseconds NPT MD simulation. Then an initial NPT equilibrium simulation (T=300 K, P=1 bar) with time step 2 fs was carried out for 20 ns. After the equilibration phase, the free energy calculations began, using the last frame of the equilibrium simulation as the starting point. We used the default MDPOW settings with five ns simulations for each alchemical free energy calculations window. The logP value obtained for glibenclamide, $logP_{md}$=4.87(0.19), corresponds to the experimental one, $logP_{exp}$=4.7[2]. Since there is no experimental logP data available for JB253, we compared the obtained value with XlogP3 parameter[3]; $logP_{md}$=5.29(0.1) and $logP_{XlogP3}$ =5.38.

Docking sulfonylureas to SUR1 protein

Docking of representative sulfonylurea ligands to close (PDB ID: 6BAA)[4] and open (PDB ID: 6C3P)[5] SUR1 was carried out using the Glide Schrödinger packet[6]. Glibenclamide (GBM) as well as other SU drugs, and both cis/trans isomers of JB253 were prepared with LigPrep and OPLS3e force field. The docking grid of 20x30x30 $Å^3$ was centred at the GBM binding site as was determined in 6BAA Cryo-EM structure[4]. All ligands were docked with a standard precision, using flexible ligand sampling.

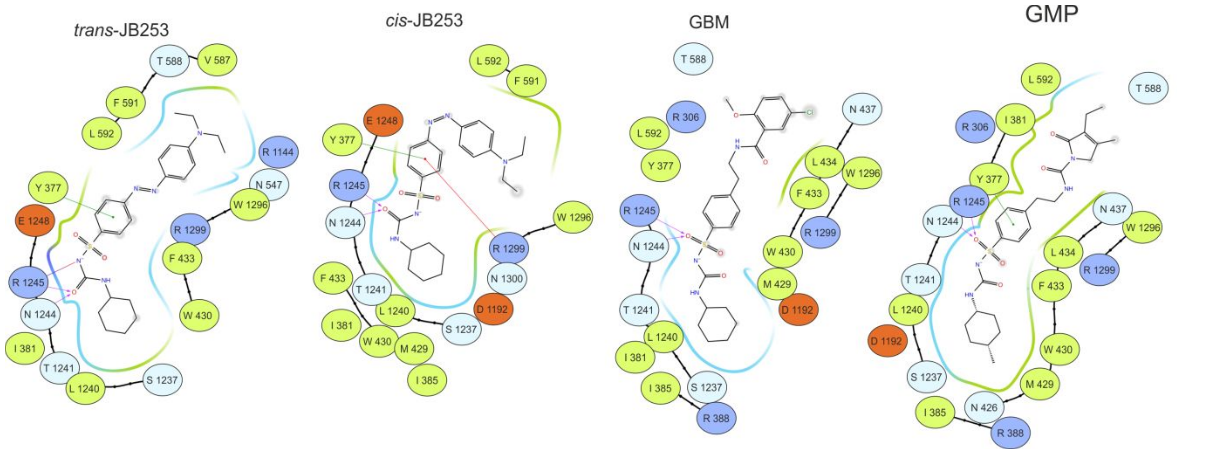

Figure S2. Schematic representation of ligand binding sites in the inward open form of SUR1. Green colour denotes for the hydrophobic residues, light blue-polar, blue-positively charged, red-negatively charged. GBM is glibenclamide, and GMP-glimepiride.

Table S1. Glide scores (in kcal/mol) of the best poses of selected ligands docked to SUR1 subunit of chimeric (rat/hamster PDB ID: 6BAA) and human (PDB ID: 6C3P) KATP channels.

Type	Ligand	SUR1 open (PDB ID:6BAA)	SUR1 closed (PDB ID:6C3P)	ΔG_{MMGBSA} [kcal/mol]	IC_{50}^{exp}
II Generation	Glibenclamide	-6.23	-6.46	-109.1	4.2 nM[7]
	Glimepiride	-7.32	-6.69	-109.9	8.3 nM[8]
	Glipizide	-6.28	-5.12	-61.0	3.8 nM[9]
	Gliclazide	-5.28	-4.09	-48.7	50 nM[7]
I Generation	Tolbutamide	-3.63	-2.53	-45.3	4.9 µM[9]
	Tolazamide	-5.86	-4.47	-45.6	NA
	Chlorpropamide	-5.97	-5.27	-42.6	NA
	Acetohexamide	-6.24	-5.77	-46.9	NA
Photo-switchable	*trans*-JB253	-5.12	-5.50	-89.1	17.6 µM[8]
	cis-JB253	-5.87	-5.01	-100.5	14.8 µM[8]
	trans-JB558	-5.13	-5.78	-89.3	37.3 µM[10]
	cis-JB558	-5.53	-6.38	-77.3	NA

Data on unbiased MD trajectories

Table S2. Average values of root-mean-square (RMSD) distances between simulated structures 20 000 data points for each 200 ns trajectory) and the starting structure (minimized and 50 ns equilibrated PDB SUR1 structures with ligands in the best docking poses).

Ligand	no	time [ns]	Cα RMSD without loops	Total Cα RMSD
APO	1	200	3.80 (0.68)	5.48 (1.06)
	2	200	2.81 (0.36)	3.88 (0.44)
	3	200	3.04 (0.50)	5.56 (0.91)
	4	200	3.90 (0.66)	5.88 (1.21)
	5	200	3.27 (0.48)	5.12 (1.00)
GBM	1	200	3.31 (0.68)	5.07 (0.80)
	2	200	3.03 (0.56)	4.81 (1.08)
	3	200	2.89 (0.40)	4.47 (0.60)
	4	200	4.34 (0.66)	5.70 (0.98)
	5	200	3.30 (0.51)	4.95 (0.66)
trans-JB253	1	200	3.27 (0.60)	4.65 (0.78)
	2	200	4.37 (0.85)	5.41 (1.10)
	3	200	3.16 (0.40)	4.88 (0.83)
	4	200	3.64 (0.62)	5.95 (0.95)
	5	200	3.31 (0.46)	4.55 (0.65)
cis- JB253	1	200	2.83 (0.39)	4.89 (0.85)
	2	200	3.32 (0.71)	4.62 (0.83)
	3	200	3.60 (0.86)	5.49 (1.14)
	4	200	3.35 (0.72)	5.12 (0.77)
	5	200	3.24 (0.53)	4.89 (0.82)

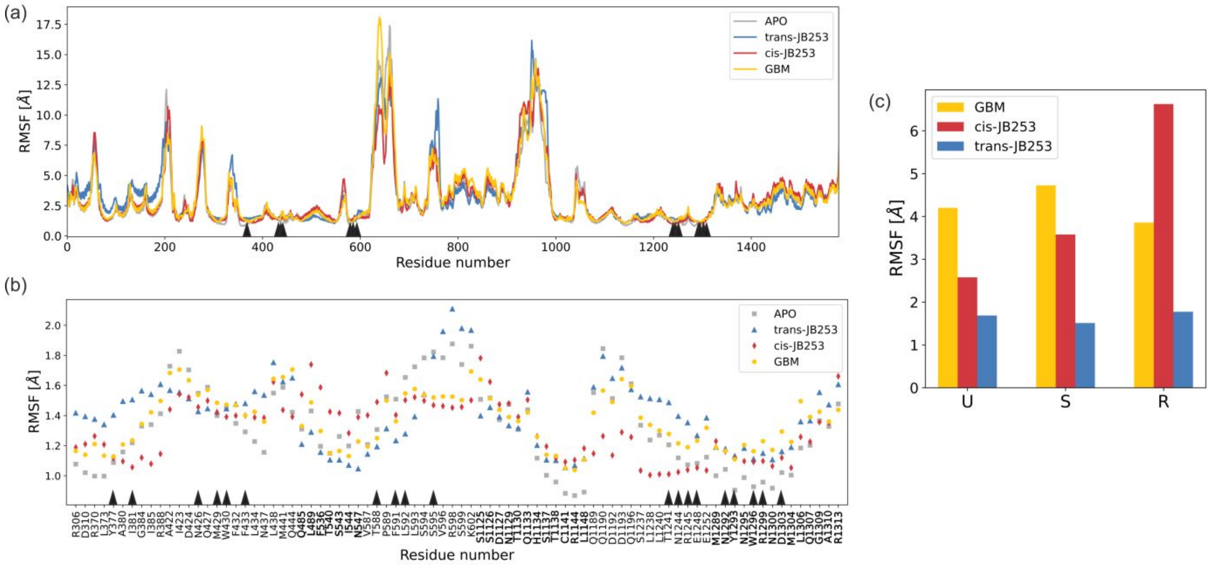

Figure S3. Values of Root-Mean-Square Fluctuation (RMSF, in Å) of residues present in SUR1 (a) and residues reaching close contacts (d < 3.5 Å with sulfonylurea ligands (b). Black arrows indicate residues which are crucial in sulfonylureas binding. Averaged RMSF of urea (U) sulfonyl (S) and the remaining part (R) of SU ligands (c).

Errors in correlation matrices

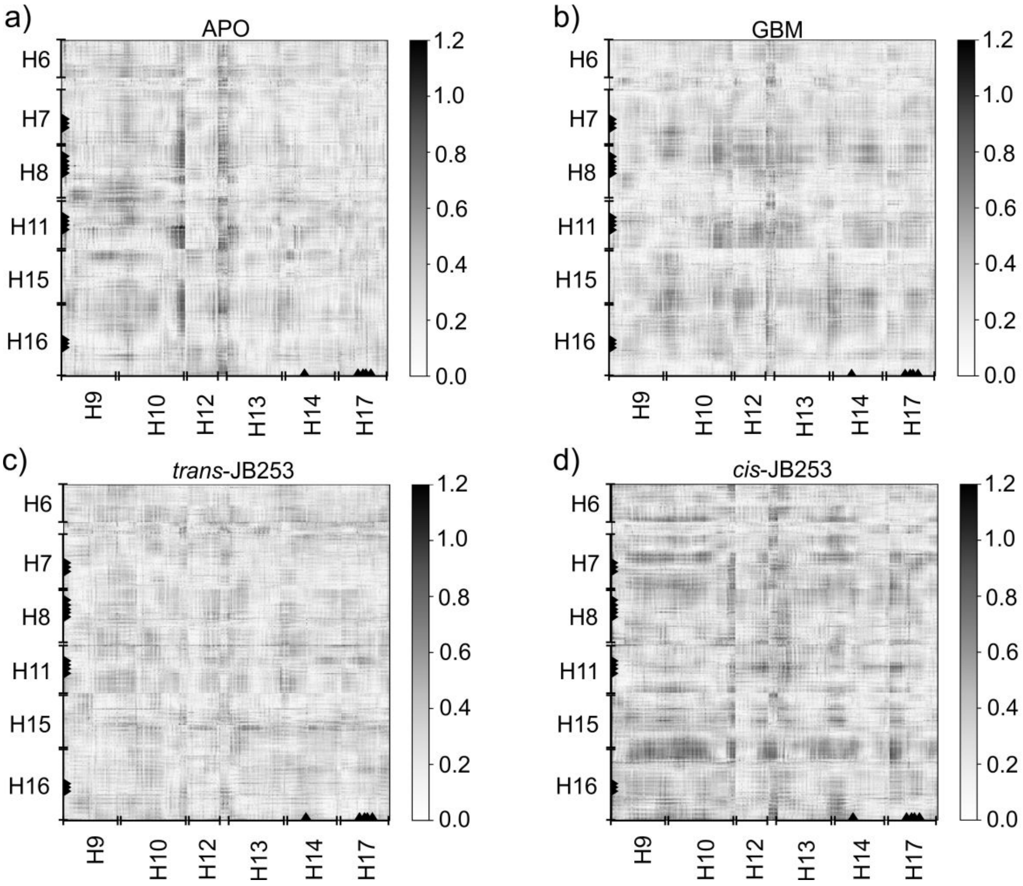

Figure S4. Errors in correlation matrices calculated from 1 µs MD trajectories data showing differences in (a) GBM and APO, (b) *cis*-JB253-SUR1 and GBM-SUR1, (c) *trans*-JB253-SUR1, (d) *cis*-JB253-SUR1 systems. The horizontal axis corresponds to L2 (helices H9, H10, H12, H13, H14, H17), while the vertical axis corresponds to helices forming L1 (H6, H7, H8, H11, H15, H16). Errors were estimated as an absolute value of a maximum difference between individual correlation matrices calculated for each 200ns trajectory. Black tringles indicate residues forming sulfonylurea binding pocket.

Water-SUR1 interactions

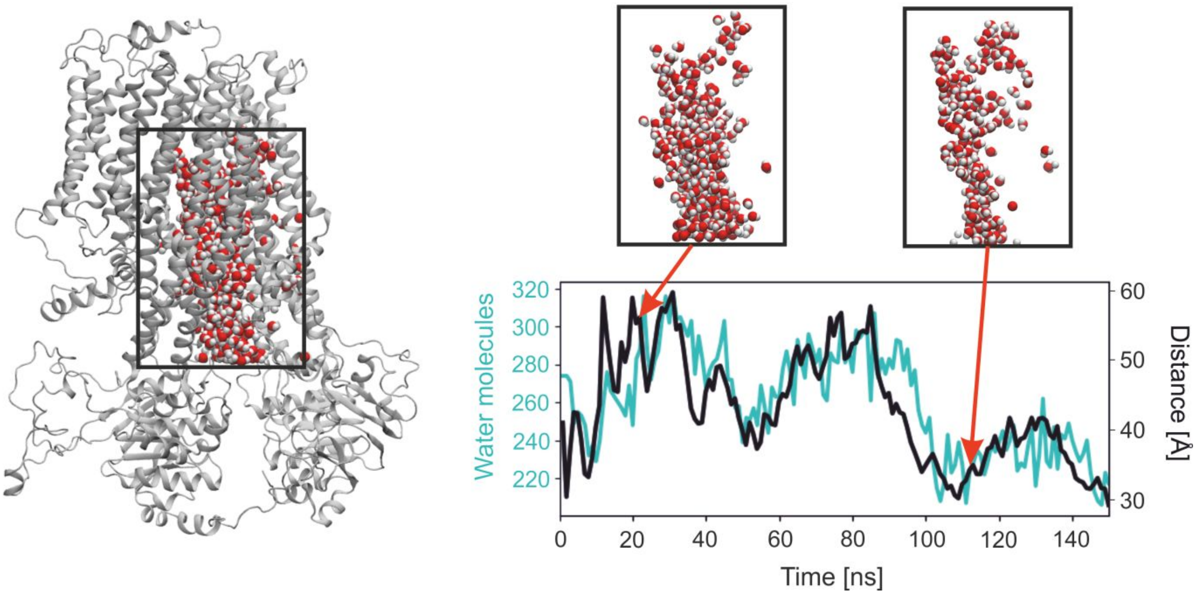

Figure S5. A number of water molecules present in L1/L2 SUR1 interface (blue) correlates with open/close states of SUR1 (distance in black). A representative snapshots from classical MD trajectories.

Table S3. Average number of water molecules present in L1/L2 SUR1 interface for each system in closed and open form. Solvent accessible area calculated for each cavity and number of water molecules which directly contact with the ligand.

System	Conformation	# of water molecules: mean (std)	Solvent accessible surface of the cavity (Å^2)	# of water molecules in contact with a ligand
APO	Open	297 (18)	50.1 (2.0)	-
	Close	244 (19)		
TRANS	Open	264 (9)	48.1 (2.4)	25 (4)
	Close	234 (14)		
CIS	Open	284 (12)	49.5 (2.8)	24 (4)
	Close	238 (13)		
GBM	Open	282 (15)	48.6 (2.2)	22 (5)
	Close	244 (17)		

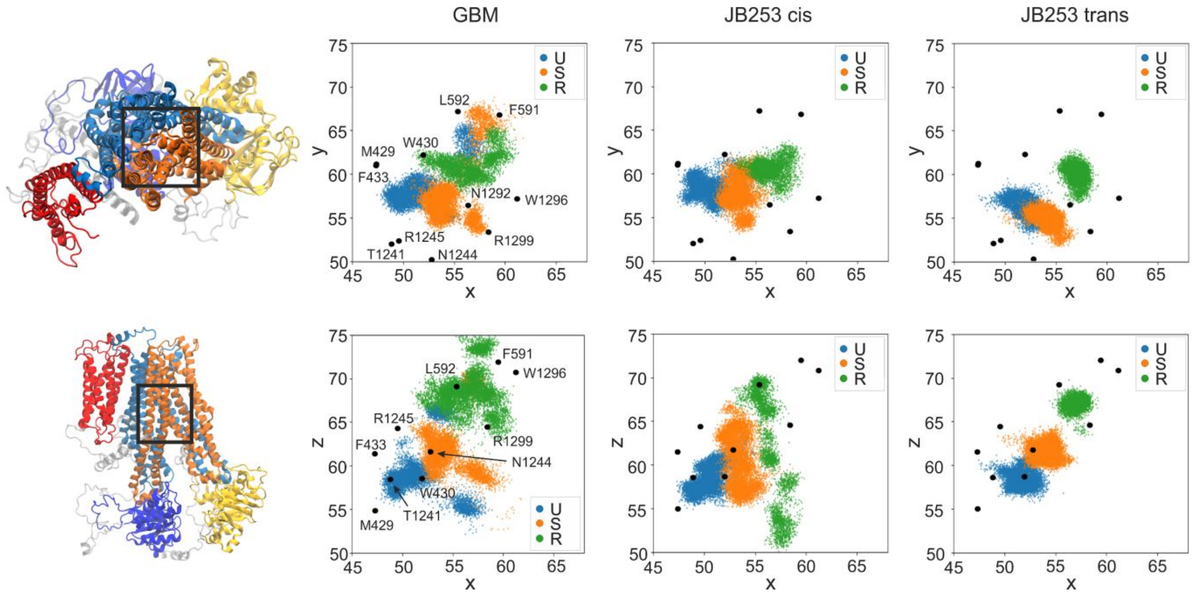

Figure S6. Positions of center of mass (COM) of each functional part of GBM and JB253 during simulation: the urea part (U), the sulfonyl group (S), and the rest of the drug (R), which is the azobenzene part in JB253 and the chloro benzamidoethyl part in GBM. With black dots the mean position (COM in the APO state) of residues which are crucial in the sulfonylureas binding are shown.

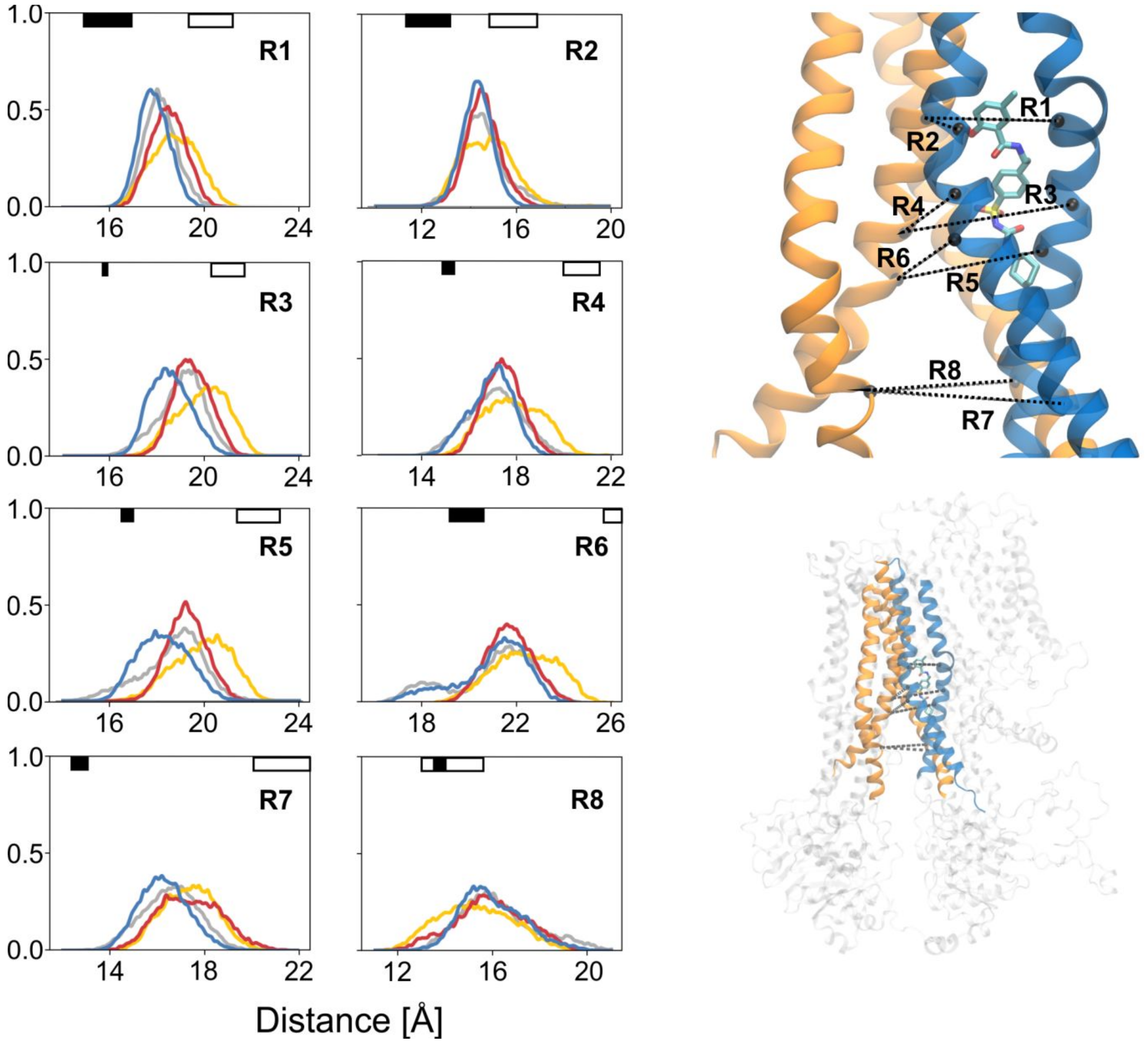

Figure S7. Distances at various points between the helices forming the SU pocket. Such data show where the drug most interferes with the natural movement of helices (compared to the APO system shown in gray). Black and white rectangles show what the values of each parameter are for closed and open Cryo-EM structures

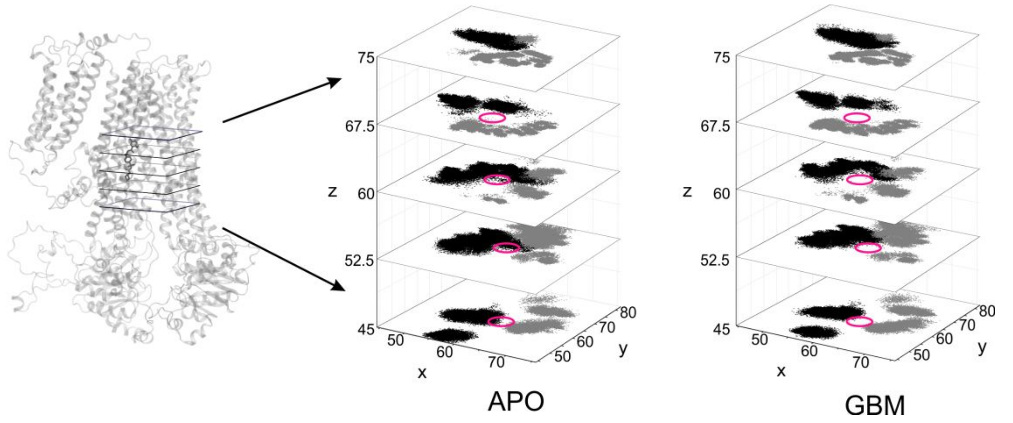

Figure S8. Tomograms of residues positions crucial for putative SUR1-KNt interactions in the APO and GBM systems. Black and gray colors represent residues from L1 and L2, respectively. Magenta circle show the most probable area of KNt penetration.

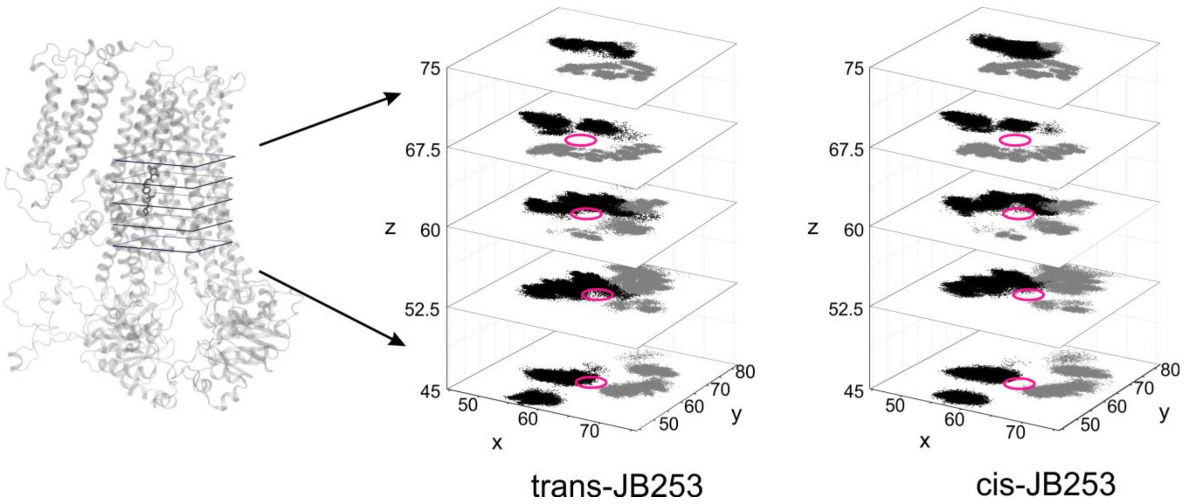

Figure S9. Tomograms of residues positions crucial for putative SUR1-KNt interactions in the APO and GBM systems. Black and gray colors represent residues from L1 and L2, respectively. Magenta circle show the most probable area of KNt penetration.

Evolutionary conservation of SUR1

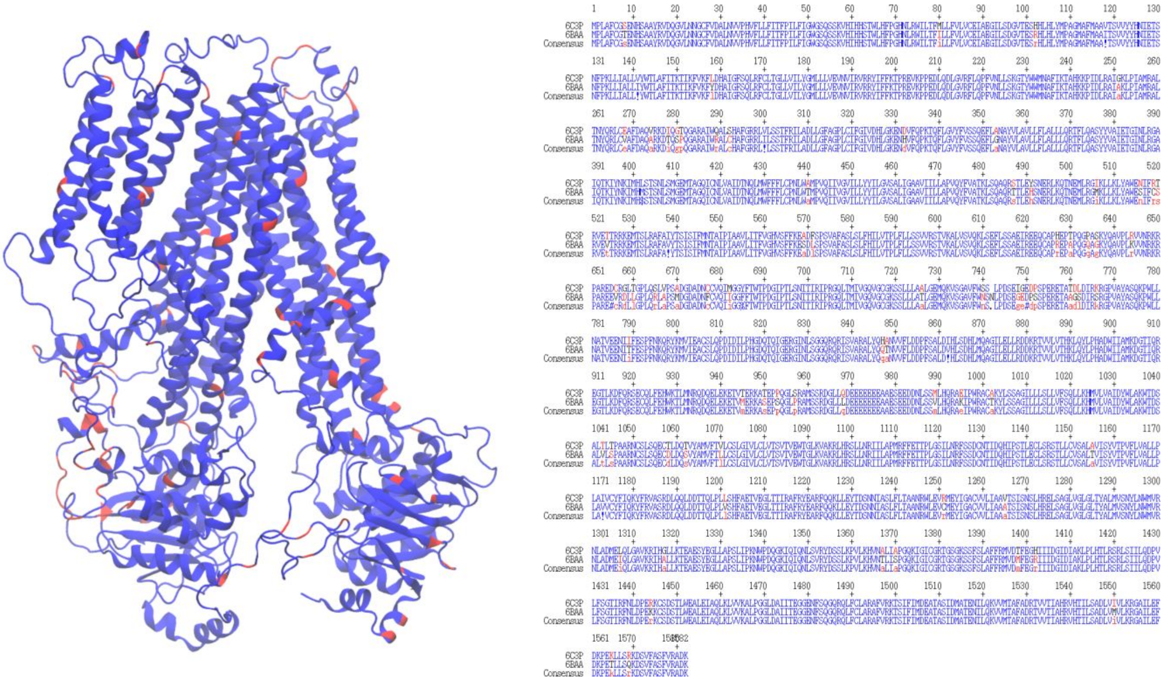

Figure S10. Comparison of sequences between hamster and human SUR1. The sequence similarity is high (95 %) and evolutionary changes are located at the surface (outskirts) of SUR1 (see red residues in the protein structure.

1. Fan, S.; Nedev, H.; Vijayan, R.; Iorga, B. I.; Beckstein, O., Precise force-field-based calculations of octanol-water partition coefficients for the SAMPL7 molecules. *Journal of Computer-Aided Molecular Design* **2021,** *35* (7), 853-870.

2. Spiller, H. A., Hypoglycemics, Oral. In *Encyclopedia of Toxicology (Third Edition)*, Wexler, P., Ed. Academic Press: Oxford, 2014; pp 989-991.

3. Cheng, T.; Zhao, Y.; Li, X.; Lin, F.; Xu, Y.; Zhang, X.; Li, Y.; Wang, R.; Lai, L., Computation of octanol-water partition coefficients by guiding an additive model with knowledge. *Journal of chemical information and modeling* **2007,** *47* (6), 2140-8.

4. Martin, G. M.; Kandasamy, B.; DiMaio, F.; Yoshioka, C.; Shyng, S.-L., Anti-diabetic drug binding site in a mammalian KATP channel revealed by Cryo-EM. *eLife* **2017,** *6*, e31054.

5. Lee, K. P. K.; Chen, J.; MacKinnon, R., Molecular structure of human KATP in complex with ATP and ADP. *eLife* **2017,** *6*, e32481.

6. Release, S., 2: Induced Fit Docking protocol. *Glide, Schrödinger, LLC, New York, NY* **2016**, 2020-2.

7. Gribble, F. M.; Tucker, S. J.; Seino, S.; Ashcroft, F. M., Tissue specificity of sulfonylureas: studies on cloned cardiac and beta-cell K (ATP) channels. *Diabetes* **1998,** *47* (9), 1412-1418.

8. Broichhagen, J.; Schönberger, M.; Cork, S. C.; Frank, J. A.; Marchetti, P.; Bugliani, M.; Shapiro, A. M. J.; Trapp, S.; Rutter, G. A.; Hodson, D. J.; Trauner, D., Optical control of insulin release using a photoswitchable sulfonylurea. *Nature Communications* **2014,** *5* (1), 5116.

9. Dörschner, H.; Brekardin, E.; Uhde, I.; Schwanstecher, C.; Schwanstecher, M., Stoichiometry of sulfonylurea-induced ATP-sensitive potassium channel closure. *Molecular pharmacology* **1999,** *55* (6), 1060-6.

10. Broichhagen, J.; Frank, J. A.; Johnston, N. R.; Mitchell, R. K.; Šmid, K.; Marchetti, P.; Bugliani, M.; Rutter, G. A.; Trauner, D.; Hodson, D. J., A red-shifted photochromic sulfonylurea for the remote control of pancreatic beta cell function. *Chemical Communications* **2015,** *51* (27), 6018-6021.
